# Neuromuscular Electrical Stimulation Enhances Lower Limb Muscle Synergies During Jumping in Martial Artists Post-Anterior Cruciate Ligament Reconstruction: A Randomized Crossover Trial

**DOI:** 10.3390/bioengineering12050535

**Published:** 2025-05-16

**Authors:** Xiaoyan Wang, Haojie Li, Jiangang Chen

**Affiliations:** 1 School of Physical Education, Hangzhou Normal University, Hangzhou 311121, China; 2 School of Exercise and Health, Shanghai University of Sport, Shanghai 200438, China; 202121070037@mail.bnu.edu.cn; 3 College of P.E. and Sports, Beijing Normal University, Beijing 100875, China; 202131070010@mail.bnu.edu.cn

**Keywords:** ACL reconstruction, neuromuscular electrical stimulation, jumping performance, muscle synergy, electromyography, martial artists

## Abstract

Objective: This study aimed to investigate the effects of neuromuscular electrical stimulation (NMES) on lower limb muscle synergies during the single-leg hop test in martial artists after anterior cruciate ligament (ACL) reconstruction. Methods: Twenty-four martial artists who underwent ACL reconstruction were recruited and performed a single-leg hop test under two conditions: with NMES (ES) and without NMES (CON). The ES condition involved using Compex SP 8.0 to deliver biphasic symmetrical wave stimulation. Jump performance metrics and electromyographic (EMG) signals were recorded. Muscle synergies of the lower limbs were extracted using non-negative matrix factorization (NMF) to analyze patterns of muscle coordination. Results: Compared with the CON condition, the ES condition significantly reduced the jump time (0.13 ± 0.05 vs. 0.18 ± 0.09; F = 5.660; *p* = 0.022) and significantly increased the contact time (0.53 ± 0.12 vs. 0.43 ± 0.21; F = 4.013; *p* = 0.049). Muscle synergy analysis revealed three distinct synergy patterns under both conditions. For synergy pattern 1, compared with the CON condition, the muscle weightings of the rectus femoris and tibialis anterior muscles were significantly increased under the ES condition (*p* < 0.001). For synergy pattern 2, compared with the CON condition, the muscle weighting of the lateral gastrocnemius muscle was significantly increased under the ES condition (*p* < 0.001). Additionally, the activation timing of synergy pattern 2 was significantly reduced under the ES condition (*p* = 0.001). Conclusion: Neuromuscular electrical stimulation enhances jump performance and alters muscle synergy patterns in martial artists after ACL reconstruction. The findings suggest that NMES can promote better lower limb muscle coordination during jumping tasks, potentially aiding in postoperative rehabilitation and performance optimization.

## 1. Introduction

Anterior cruciate ligament (ACL) injuries are becoming increasingly prevalent among athletes, especially in those sports that engage in high-intensity, rapid change-of-direction movements, such as basketball, football, skiing, and wushu [1,2]. ACL injuries not only result in the inability of athletes to continue to participate in competitive activities but may also affect their athletic performance and recovery [3]. Studies have shown that ACL injuries can severely affect athletes’ stability, explosive power, jumping ability, and execution of motor skills [4]. Moreover, athletes with ACL injuries have a significant decrease in lower limb strength, especially during one-legged jumps and changes in direction, leading to a significant decrease in athletic performance [5]. Another study pointed out that athletes’ control, quick reaction ability, and power output are significantly reduced after ACL injury, which makes athletes prone to secondary injuries or unable to return to their pre-injury level of competition during high-intensity exercise [6]. Therefore, ACL injuries have adverse effects on the overall athletic performance of athletes, especially in sports that require a high degree of explosive power and stability [7].

In contrast, wushu, as a sport requiring a high degree of agility, explosiveness, and coordination, has movement requirements that include a rapid change in direction, jumping, and complex technical maneuvers [8]. Among them, the one-legged jumping ability is one of the crucial abilities of wushu athletes in training and competition, and jumping movements in wushu are not only competition skills, but also involve tactics and critical moments in the competition [9]. For example, in wushu competitions, it is also often necessary to land quickly and regain posture for defense or counterattack. Previously, it has been shown that the ability to jump on one foot directly affects the mobility of wushu athletes in competition. The stability and explosive power of one-legged jumps not only affect the athletes’ jumping height and distance but also determine whether they can accurately complete difficult landing maneuvers [10,11]. Therefore, strengthening the one-legged jumping ability of wushu athletes is of great significance for improving performance and preventing sports injuries.

Muscle synergy refers to the collaborative firing of multiple muscle groups in the same movement to achieve the efficient completion of the motor task [12]. In jumping, muscle synergy is crucial for athletes’ jump height, explosive power, and stability [13]. The effectiveness of muscle synergy enables athletes to perform efficient locomotor movements with less energy expenditure, reducing the risk of injury [14]. Studies have shown that the jumping process, especially single-leg jumping, involves the coordinated firing of several lower limb muscle groups (e.g., quadriceps, gastrocnemius, and hip flexors) [15]. Electromyography (EMG) signals and non-negative matrix factorization (NMF) are tools commonly used to analyze muscle synergies and can help to identify patterns of muscle group cooperation in complex motor tasks [16]. For one-legged jumps, the complexity of muscle synergies is more prominent than for two-legged jumps, as athletes need to support their body weight and generate sufficient thrust with one leg alone [17]. However, despite the large number of studies on bipedal jumping and muscle synergy, there are still relatively few studies on muscle synergy in one-legged jumping. Most of the existing studies have focused on muscle synergy during bipedal force generation, and the pattern of muscle synergy involved in one-legged jumping is different from it, and thus needs to be further explored in depth. In addition, neuromuscular electrical stimulation (NMES) is a technique that stimulates nerve conduction through electric currents and triggers muscle contraction, and it is widely used in rehabilitation and sports performance enhancement [18]. Studies have shown that NMES can effectively promote the strength, endurance, and synergy of lower limb muscles, thus improving sports performance [19,20]. In postoperative knee rehabilitation, NMES has been found to help patients regain knee muscle strength and improve athletic performance [21]. Although some studies have confirmed the effectiveness of NMES in improving lower limb strength and athletic performance, there is uncertainty about whether it is effective in improving intermuscular synergy, especially in complex jumping maneuvers. Most of the current studies have focused on strength and endurance gains, and further exploration is still needed on how NMES affects the synergistic activity of lower limb muscles, especially in one-legged jumping.

In recent years, neuromuscular electrical stimulation (NMES) has demonstrated significant potential as a non-invasive intervention in the field of motor rehabilitation and performance enhancement. Numerous studies have shown that NMES can act through dual mechanisms: on the one hand, it enhances muscle contraction through the direct activation of motor neurons [22], and on the other hand, it optimizes neural control strategies by modulating spinal cord and cortical excitability [23]. Electromyography (EMG) technology provides a key tool for revealing these mechanisms, and muscle activation patterns recorded by surface EMG can be used to quantitatively assess the effects of NMES intervention [24]. Particularly in the field of ACL rehabilitation, previous studies have found that NMES combined with centrifugal training significantly improves quadriceps activation; recent studies have further demonstrated that NMES optimizes muscle activation timing in athletes, and these findings were validated by EMG muscle synergy analysis [25]. However, most of the existing studies focus on simple movements or the general population, and there is still a knowledge gap for specialized sports that require complex neuromuscular control (e.g., wushu single-leg jumping). In this study, by integrating NMES intervention with high-density EMG monitoring, we systematically assessed its effects on muscle synergy patterns in postoperative ACL wushu athletes for the first time, providing new evidence for understanding the role of NMES in the rehabilitation of specific motor skills.

This study makes several innovative contributions to the field of neuromuscular electrical stimulation (NMES), significantly advancing the depth and breadth of existing research. First, in terms of study population, we focused our NMES intervention for the first time on a group of martial arts athletes undergoing ACL reconstruction. This group has unique athletic demands, with its high explosive power, frequent single-leg jumps, and complex landing cushions [26], making it often difficult for traditional rehabilitation programs to meet their specialized needs. Unlike previous NMES studies that mainly focused on the general population or athletes in traditional sports [27], our work fills the gap in the application of NMES in the rehabilitation of high-demand specialized sports. Second, in terms of research methodology, we innovatively applied the non-negative matrix factorization (NMF) technique to muscle synergy analysis, which provides a new perspective to reveal the neuromodulator mechanisms of NMES. While traditional NMES studies are mostly limited to the measurement of muscle strength or sport performance indexes [28], we quantified for the first time the effect of NMES on the muscle synergy patterns of the lower limbs by the NMF method, and found that it not only enhances the synergistic activation of the rectus femoris and anterior tibialis muscles (optimizing jumping explosiveness), but also improves the contribution of the lateral head of gastrocnemius muscle and shortens the time of its activation (improving landing cushioning ability); these findings provide a new perspective for understanding the mechanisms by which NMES promotes neuroplasticity [29]. Third, in terms of study design, we used a randomized crossover trial protocol to effectively control for the effects of individual differences by subjecting the same subjects to both NMES and control conditions, a rigorous design that is quite innovative in NMES research. Finally, in terms of theoretical contributions, our study goes beyond the traditional rehabilitation paradigm that emphasizes on muscle strength recovery, and suggests for the first time that NMES may optimize the overall movement pattern by remodeling the spinal cord or cortical motor control strategies, which introduces the concept of “neuromuscular synergistic optimization” for ACL postoperative rehabilitation. These innovations not only expand the depth of NMES research but also provide an important theoretical basis and practical guidance for the postoperative rehabilitation practice of martial arts athletes and other special groups.

Although there have been studies focusing on the role of traditional strength training on rehabilitation after ACL reconstruction, there are fewer studies addressing the effects of neuromuscular electrical stimulation (NMES) on one-legged jumping and muscle synergy in martial arts athletes. Wushu requires athletes to have a high level of one-legged jumping and precise muscle control. In this study, we investigated the effects of neuromuscular electrical stimulation (NMES) on one-legged jumping ability and muscle coordination in a specific group of wushu athletes. Although traditional strength training has a role in improving athletes’ strength and explosive power, the improvement of one-legged jumping ability relies more on muscle coordination and motor control, which has not been sufficiently emphasized in existing studies. By introducing NMES as a novel intervention, the present study not only helped wushu athletes to improve the strength of lower limb muscles, but also potentially facilitated the coordination between muscles and optimized the performance of one-legged jumping. Particularly in the rehabilitation process after ACL reconstruction, this intervention may become an effective means to promote synergistic muscle recovery and improve jumping performance, thus providing new ideas for the recovery and training of wushu athletes.

## 2. Methods

### 2.1. Participants

Given the exploratory nature of this study, an a priori power analysis was conducted using G*Power software. The sample size was calculated using GPower 3.1, with α = 0.05, 1 − β = 0.80, and a small effect size (d = 0.2) based on pilot data and prior NMES research [30]. This yielded N = 20. To account for potential attrition, 24 martial arts athletes (aged 18–30 years) who had undergone anterior cruciate ligament (ACL) reconstruction were recruited. This study was ethically approved by Beijing Normal University (Approval number: BNU20241125). All subjects signed an informed consent form.

Participants were required to meet inclusion criteria including the following: (1) ≥10 years of specialized martial arts training with top-five provincial competition placements in the preceding three years; (2) MRI-confirmed unilateral isolated ACL rupture treated via arthroscopic single-bundle autograft reconstruction; (3) completion of a 3-month standardized postoperative rehabilitation protocol (progressive resistance training, balance exercises, and gait re-education) without electrical stimulation; and (4) postoperative Lysholm score ≥85 with full knee range of motion (0° extension–135° flexion). Exclusion criteria included the following: (1) concurrent meniscal grade III tears, cartilage defects > ICRS grade 2, or collateral ligament injuries; (2) neurological disorders (e.g., multiple sclerosis or spinal cord injury), metabolic myopathies, or rheumatoid arthritis; (3) recent (≤6 months) use of muscle relaxants, corticosteroids, or neuroblocking agents; and (4) cardiac pacemakers, epilepsy, or electrostimulation hypersensitivity. Demographic details are presented in Table 1.

### 2.2. Experimental Design

A randomized crossover design was implemented to investigate the effects of neuromuscular electrical stimulation (ES) on muscle synergies in ACL-reconstructed martial artists. Participants completed three months of standard physiotherapy prior to the study. During the fourth postoperative month, they were randomly assigned to receive either ES or sham stimulation (control condition, CON), with a 4-day washout period between conditions.

Intervention Protocol:

1. Washout Phas: 4-day interval with prohibition of strenuous activity.
2. Warm-up Protocol:

10 min stationary cycling (50 W load, 60 rpm).

10 min dynamic stretching (3 sets × 30 s for quadriceps, hamstrings, and gastrocnemii).

3. Stimulation Delivery:

ES Group: Functional electrical stimulation synchronized with stance phase.

CON Group: Identical device activation without current output.

4. Immediate Testing: Biomechanical assessments commenced within 5 min post-intervention.

### 2.3. Electrical Stimulation Parameters

The neuromuscular activation of the quadriceps was achieved using a Compex SP 8.0 stimulator (Compex Medical SA, Geneva, Switzerland) with the following settings (Table 2):

### 2.4. Single-Leg Hop Test

**Figure 1:**
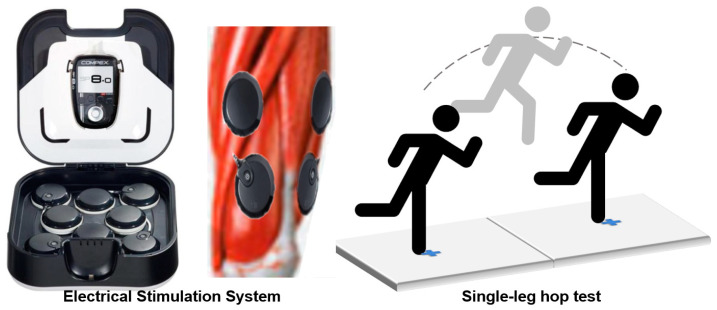
Compex SP 8.0 electrical stimulation system.

Lower limb functional performance was quantified using a Kistler 9287CA force plate (1000 Hz sampling rate). Testing procedures included the following (Figure 1):

1. Barefoot single-leg stance on force plate center.
2. Maximal forward horizontal jump with arms crossed.
3. Landing stabilization (≥3 s visual assessment) for data validity.
4. Three trials (2 min rest intervals), with optimal trial retained for analysis.

Primary Metrics:

1. Jump Time (JT): Duration from the ground reaction force returning to 0 to its appearance and drastic increase.
2. Maximum Vertical Force (MVF): Peak force normalized to body weight (BW).
3. Contact Time (CT): Duration from the drastic increase in the ground reaction force after it returns to 0 until it returns to the stable value again.

### 2.5. Electromyography (EMG) Data Acquisition

**Figure 2:**
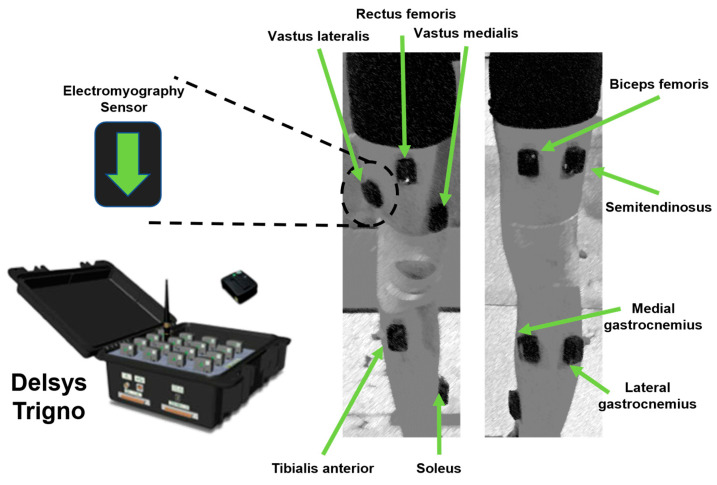
Surface EMG testing and analysis system.

The Delsys surface electromyography device was used to collect the EMG signals of 9 muscles on the side of ACL reconstruction, namely, the vastus medialis (VM), vastus lateralis (VL), rectus femoris (RF), biceps femoris (BF), semitendinosus (ST), tibialis anterior (TA), medial gastrocnemius (MG), lateral gastrocnemius (LG), and soleus (SOL). The skin was cleaned before the test, and the electrodes were fixed with tape to ensure stability. The electrode positioning followed the SENIAM guidelines. The EMG data of the injured side leg during the participants’ jumps were collected, and muscle synergy was analyzed through non-negative matrix factorization (Figure 2).

### 2.6. Muscle Synergy Computation

EMG processing and synergy extraction were performed in R (v4.2.0; musclesyneRgies v1.2.5) using the following pipeline:

1. Signal Conditioning:

Bandpass filtering (20–400 Hz, 4th-order Butterworth).

Full-wave rectification.

Low-pass filtering (20 Hz cutoff, 4th-order Butterworth).

Time normalization to 200 data points.

2. Non-negative Matrix Factorization (NMF):

For the decomposition of muscle activation patterns *D*(*t*) into time-invariant synergy vectors *Wi* and activation coefficients *Ci*(*t*), the optimal synergy number was defined as the minimum required to achieve ≥90% variance accounted for (VAF).

3. Pattern Matching:

Reference synergies from control conditions were clustered via k-means. Experimental synergies with Pearson’s *r* > 0.6 against reference templates were classified as homologous.

4. Quantified Parameters:

Total synergy patterns;

Muscle weighting coefficients (*Wi*);

Activation duration (*Ci*(*t*) > 0.3).

### 2.7. Statistical Analysis

A three-factor linear mixed-effects model was used for analysis, which included two fixed factors (test condition and test stage) and one random factor (subject ID). This study mainly explored the main effects of the test condition (electrical stimulation vs. control) and the test stage (first stage vs. second stage), and no interaction effect was found. *p* < 0.05 was used as the standard for statistical significance. The data analysis was completed using SPSS 26.0 software (SPSS Inc., Chicago, IL, USA).

## 3. Results

Table 3 presents the kinematic results of the single-leg hop test. The study revealed that in all the indicators of single-leg jumps, the main effect of time was not significant (*p* > 0.05). However, for jump time and contact time, the condition effect was significant (*p* < 0.05). Specifically, the take-off time under the electrical stimulation (ES) condition was significantly shorter than that of the control group (CON) (F = 5.660; *p* = 0.022). This result indicates that the ES group had a faster neuromuscular conduction speed. Meanwhile, the landing time under the ES condition was significantly longer than that of the CON group (F = 4.013; *p* = 0.049), suggesting that the ES group had a better landing cushioning performance.

**Figure 3:**
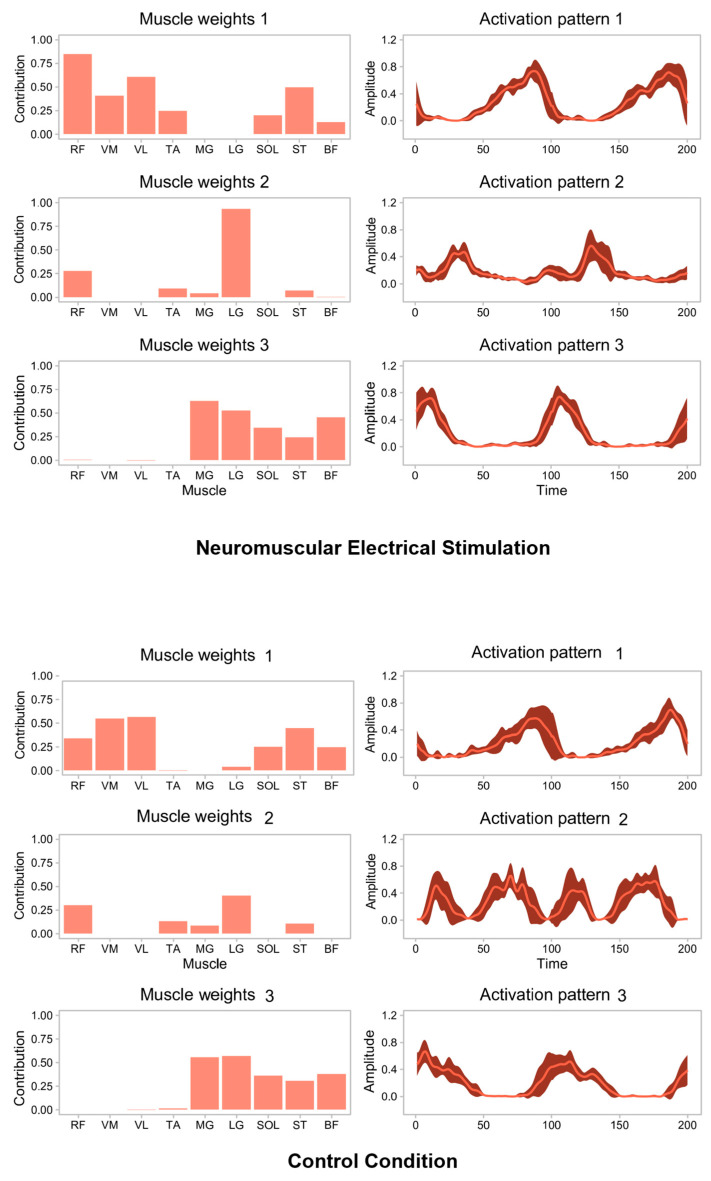
Muscle synergy patterns under different conditions.

As can be seen from Figure 3, three muscle synergy patterns were identified in both the ES group and the CON group. Based on this, an in-depth analysis was further carried out on the differences in muscle weights and muscle activation times within each muscle synergy pattern.

The results in Table 4 show that in muscle synergy pattern 1, compared with the CON condition, the muscle weights of the rectus femoris (F = 34.865; *p* = 0.000) and tibialis anterior muscles (F = 82.980; *p* = 0.000) under the ES condition increased significantly. Table 5 shows that for muscle synergy pattern 2, the muscle weight of the lateral gastrocnemius muscle under the ES condition increased significantly compared with the CON condition (F = 84.390; *p* = 0.000). However, the results in Table 6 indicate that in muscle synergy pattern 3, there was no significant difference in muscle weights between the CON condition and the ES condition. In addition, the data in Table 7 show that compared with the CON condition, the activation time of muscle synergy pattern 2 under the ES condition was significantly shortened (F = 13.407; *p* = 0.001).

## 4. Discussion

In this study, we found that neuromuscular electrical stimulation (NMES) significantly affected the jumping ability of wushu athletes after ACL reconstruction, specifically in terms of shorter jumping time and longer landing time. According to the results of the single-leg jump test, the jumping time of the NMES group was significantly shorter than that of the control group (CON), a result that suggests that NMES significantly improves the speed of neural response and shortens the preparation time from the resting state to the jump [31]. By accelerating the transmission of neural signals, NMES helps to improve the neuromuscular response of the athlete, thus optimizing the swiftness and explosiveness of the jumping action [32]. This improved reaction speed is important for the explosive athletic ability of athletes during recovery, especially in sports that require rapid reaction and explosive power, and can promote the athletes’ postoperative athletic performance.

Meanwhile, landing time was significantly longer in the NMES condition than in the control group, suggesting that NMES improved athletes’ landing cushioning ability. The longer contact time meant that during landing, athletes in the NMES group were able to absorb and disperse impact forces more efficiently, thereby reducing the burden on the joints. This increased cushioning capacity reduces the risk of secondary injuries due to unstable landings, and for high-intensity athletes, especially those who have undergone ACL reconstructive surgery, good landing control is crucial, not only to help reduce the risk of re-injury, but also to improve athletes’ performance and consistency in actual competition [33].

Our findings are in line with some previous studies, which have shown that NMES can facilitate athletes’ motor performance and force control during jumping and landing by improving muscle activation patterns and coordination [34]. In addition, it has been shown that NMES enhances the activation of muscles around the knee joint, which in turn improves lower limb strength and athletic performance [35]. Our study further confirmed the role of NMES in enhancing jump reaction speed and landing cushioning ability, especially in athletes after ACL reconstruction, a finding that further demonstrates the role of NMES as a rehabilitative tool for postoperative recovery. This study analyzed in depth the effects of neuromuscular electrical stimulation (NMES) on lower limb muscle synergism in wushu athletes after ACL reconstruction of the knee. The results of the study showed that neuromuscular electrical stimulation significantly enhanced the synergy between the quadriceps and calf muscle groups (e.g., rectus femoris and tibialis anterior) and improved the athletes’ jumping ability, neural response, and cushioning in several ways. This suggests that NMES plays an important role in enhancing the level of activation of these key muscle groups, contributing to improved muscle explosiveness and stability in jumping [36]. In addition, the muscle weights of the lateral gastrocnemius (F = 84.390; *p* = 0.000) were significantly increased in the NMES condition in muscle synergy mode 2, further demonstrating the enhanced synergy of the calf muscle groups in response to NMES. This enhanced synergy may help athletes to better control ground contact during jumping, which in turn enhances jump height and explosive power [37]. However, in muscle synergy mode 3, we did not observe a significant difference in muscle weights between the NMES and CON conditions, suggesting that the effect of NMES on some muscle groups may not be as significant as on the quadriceps and calf muscle groups. Nevertheless, the activation time in muscle synergy mode 2 was significantly shorter in the NMES condition (F = 13.407; *p* = 0.001), suggesting that NMES not only enhances muscle activation, but also improves athletes’ muscular reaction speed, thus facilitating athletes’ neuromuscular coordination, especially during dynamic movements [38].

Firstly, these results are consistent with previous research, which has shown that neuromuscular electrical stimulation helps to increase the activation level of target muscle groups and improve muscle synergy [39]. Previous evidence found that neuromuscular electrical stimulation significantly improved activation levels of the quadriceps and biceps femoris muscles and enhanced muscle coordination in patients recovering from knee surgery [40]. It has also been noted that NMES can help promote coordination between the nervous system and the muscular system, especially when performing explosive demanding sports, which is crucial for recovery and performance enhancement in athletes [41]. Our study similarly found that NMES significantly increased the activation of quadriceps and calf muscle groups (e.g., tibialis anterior and gastrocnemius) and enhanced synergistic effects between them. These consistent results suggest that neuromuscular electrical stimulation serves as an effective rehabilitation tool to help athletes overcome muscle imbalances and delayed neural responses during the recovery process, thereby accelerating the rehabilitation process and improving athletic performance.

In addition, no significant difference was found between NMES and control group in muscle synergy mode 3. This suggests that although NMES can enhance synergistic effects in some muscle groups, its effects may vary depending on the muscle type or the specific movement pattern. In some cases, NMES may not activate specific muscle groups (e.g., posterior thigh muscle group or gastrocnemius) as significantly as the quadriceps or anterior calf muscle groups. This result suggests that future research could further explore the differences in response to NMES across muscle types, particularly which muscle groups benefit more from NMES during complex motor tasks. It is also possible that the stimulus intensity, frequency, or duration of NMES may not be sufficiently modulated to an optimal level in certain muscle synergy modes, resulting in less impact on certain muscle groups.

In addition, it was found that NMES significantly shortened the time of muscle activation in muscle synergistic mode 2, suggesting that NMES not only enhances the intensity of muscle activation, but also improves the speed of muscle response, which promotes the enhancement of neuromuscular coordination in athletes [42]. Enhanced neural responses are critical for athlete recovery, especially in complex and rapidly changing sport environments [43]. Therefore, this elevated reaction speed and neural coordination helps athletes adapt more quickly to various sport loads after recovery, especially in high-intensity sports that require quick reactions and precise control, and reduces the risk of sport injuries [44]. Studies have shown that the enhancement of neural response is closely related to the improvement of sports performance, especially in the areas of explosive power and power output [45]. This further validates the potential of NMES to help athletes recover and improve athletic performance by promoting improved neural responses during training and rehabilitation.

Consistently with previous findings, our study demonstrated that neuromuscular electrical stimulation has an important role in promoting muscle synergy. For example, previous studies have found that neuromuscular electrical stimulation enhances the coordination of limb muscles, thereby enhancing athletic performance and functional recovery [46]. However, the present study also found some unique results, such as a significant increase in the muscle weight of the lateral gastrocnemius muscle in synergistic mode 2. This finding suggests that the involvement of the lateral gastrocnemius muscle is particularly important for the recovery of jumping ability, possibly reflecting its key role in ground bounce and bouncing processes. In addition, the reduction in activation time further suggests that with the help of NMES, athletes were able to mobilize lower limb muscle groups more rapidly, with an improved response rate in the calf muscles in particular. This result is in line with previous studies which have shown that neuromuscular electrical stimulation improves athletes’ reaction speed and the timeliness of power output [47]. Through these changes, athletes can gain better control and performance in explosive movements such as jumping, leading to greater advantage in training and competition.

The optimization of muscle synergy patterns found in the present study (e.g., the synergistic enhancement of rectus femoris and tibialis anterior muscles and the improved timing of gastrocnemius activation) may reflect NMES-induced alterations in multilevel neural adaptations. Recent studies have shown that NMES not only enhances muscle strength through peripheral mechanisms (e.g., increased motor unit recruitment) but also optimizes motor control by modulating corticospinal excitability and motor cortex plasticity [48]. Specifically, the shorter jumping time (reflecting elevated nerve conduction velocity) observed in the present study is consistent with findings from previous studies, which confirmed that NMES accelerates force generation by increasing the efficiency of signaling between the motor cortex and the spinal cord [49]. More importantly, changes in muscle synergy patterns (e.g., the shortening of gastrocnemius activation timing in synergistic mode 2) may correspond to the remodeling of neural circuits in the spinal cord layers by NMES, where regular electrical stimulation strengthens synaptic connections between class Ia afferent fibers and α-motor neurons to optimize the activation timing of multiple muscle groups [50]. This mechanism fits with motor learning theory: NMES may facilitate the formation of more efficient motor programs in the CNS by providing repetitive, temporally precise afferent feedback [51]. In addition, it was found that NMES enhances dynamic balance in patients with chronic ankle instability, and its mechanism of action is being related to the improvement of the timing of reactivation of the calf triceps [52]. Together, this evidence supports the conclusion of the present study that the enhancement of jumping performance by NMES in martial arts athletes not only stems from the enhancement of muscle strength but also relies on its optimization of the neuromuscular control system, and that this dual action makes it an ideal intervention for the postoperative rehabilitation of ACL.

## 5. Conclusions

**Neuromuscular** electrical stimulation (NMES) significantly improved jumping performance in martial arts athletes during rehabilitation after ACL reconstruction, particularly demonstrating shorter jumping times and longer landing times in the single-leg jump test. This result suggests that NMES is effective in increasing neural reaction speed and optimizing muscle synergy, thereby promoting the recovery of motor function and enhancing performance. Especially in the early stages of postoperative rehabilitation, NMES can be used as a powerful aid to help athletes recover faster and provide new ideas and methods for the training of martial arts athletes.

## Figures and Tables

**Table 1:** Demographic information of participants.

Indicator	(n = 24)
Gender	50% female
Age (years)	25.7 ± 4.6
Height (cm)	172.8 ± 8.9
Weight (kg)	67.3 ± 9.6
Training Experience (years)	13.7 ± 4.6

**Table 2:** Electrical stimulation parameters.

Parameter	ES Condition	Control Condition
Waveform	Biphasic symmetric square	Biphasic symmetric square
Frequency	100 Hz	3 Hz
Pulse Width	300 µs	300 µs
Intensity	25–35 mA (individual pain threshold)	1 mA (subsensory)
Duration	15 min	15 min
Electrode Placement	Quadriceps motor points	Identical to ES

**Table 3:** Single-leg hop test kinetics.

			Test Condition		Test Stage	
	ES	CON	F	*p*	**F**	***p***
Jump Time	0.13 ± 0.05	0.18 ± 0.09	5.660	0.022	0.026	0.874
MVF	210.12 ± 55.67	212.65 ± 78.89	0.017	0.898	0.183	0.673
Contact Time	0.53 ± 0.12	0.43 ± 0.21	4.013	0.049	1.272	0.234

**Table 4:** Comparison of muscle weights in synergy pattern 1 under different conditions.

			Test Condition		Test Stage	
	ES	CON	F	*p*	**F**	***p***
RF	0.81 ± 0.31	0.33 ± 0.25	34.865	0.000	0.000	0.983
VM	0.39 ± 0.23	0.54 ± 0.31	3.624	0.063	0.078	0.783
VL	0.58 ± 0.18	0.56 ± 0.19	0.140	0.710	0.180	0.675
TA	0.24 ± 0.12	0.01 ± 0.03	82.980	0.000	0.084	0.774
MG	0.01 ± 0.03	0.01 ± 0.02	0.000	1.000	1.134	0.298
LG	0.01 ± 0.02	0.01 ± 0.01	0.000	1.000	0.481	0.495
SOL	0.20 ± 0.15	0.24 ± 0.14	0.912	0.345	0.226	0.639
ST	0.48 ± 0.13	0.43 ± 0.14	1.644	0.206	0.337	0.567
BF	0.14 ± 0.06	0.23 ± 0.15	4.033	0.051	2.549	0.124

Note: VM: vastus medialis; VL: vastus lateralis; RF: rectus femoris; BF: biceps femoris; ST: semitendinosus; TA: tibialis anterior; MG: medial gastrocnemius; LG: lateral gastrocnemius; SOL: soleus.

**Table 5:** Comparison of muscle weights in synergy pattern 2 under different conditions.

			Test Condition		Test Stage	
	ES	CON	F	*p*	**F**	***p***
RF	0.27 ± 0.09	0.29 ± 0.11	0.475	0.494	0.275	0.605
VM	0.00 ± 0.01	0.00 ± 0.01	0.000	1.000	0.062	0.805
VL	0.00 ± 0.02	0.00 ± 0.01	0.000	1.000	0.399	0.534
TA	0.09 ± 0.07	0.12 ± 0.04	3.323	0.075	0.090	0.767
MG	0.03 ± 0.04	0.05 ± 0.04	3.000	0.090	0.355	0.557
LG	0.91 ± 0.25	0.39 ± 0.12	84.390	0.000	0.005	0.942
SOL	0.00 ± 0.01	0.00 ± 0.05	0.000	1.000	0.003	0.957
ST	0.08 ± 0.04	0.08 ± 0.06	0.000	1.000	0.009	0.925
BF	0.00 ± 0.03	0.00 ± 0.04	0.000	1.000	0.082	0.777

Note: VM: vastus medialis; VL: vastus lateralis; RF: rectus femoris; BF: biceps femoris; ST: semitendinosus; TA: tibialis anterior; MG: medial gastrocnemius; LG: lateral gastrocnemius; SOL: soleus.

**Table 6:** Comparison of muscle weights in synergy pattern 3 under different conditions.

			Test Condition		Test Stage	
	ES	CON	**F**	***p***	**F**	***p***
RF	0.00 ± 0.01	0.00 ± 0.03	0.000	1.000	0.003	0.958
VM	0.01 ± 0.03	0.01 ± 0.02	0.000	1.000	0.008	0.929
VL	0.00 ± 0.02	0.00 ± 0.04	0.000	1.000	0.073	0.790
TA	0.01 ± 0.04	0.01 ± 0.01	0.000	1.000	0.025	0.876
MG	0.61 ± 0.35	0.55 ± 0.25	0.467	0.498	0.103	0.751
LG	0.51 ± 0.26	0.55 ± 0.29	0.253	0.617	0.263	0.613
SOL	0.33 ± 0.16	0.35 ± 0.17	0.176	0.677	0.114	0.739
ST	0.22 ± 0.18	0.30 ± 0.19	2.242	0.141	0.270	0.608
BF	0.44 ± 0.22	0.36 ± 0.15	2.166	0.148	0.078	0.783

Note: VM: vastus medialis; VL: vastus lateralis; RF: rectus femoris; BF: biceps femoris; ST: semitendinosus; TA: tibialis anterior; MG: medial gastrocnemius; LG: lateral gastrocnemius; SOL: soleus.

**Table 7:** Comparison of activation time of each muscle synergy pattern in different conditions.

			Test Condition		Test Stage	
	ES	CON	**F**	***p***	**F**	***p***
Synergy 1	81 ± 28	78 ± 31	0.124	0.727	0.027	0.809
Synergy 2	53 ± 24	80 ± 27	13.407	0.001	0.330	0.571
Synergy 3	54 ± 21	58 ± 19	0.479	0.492	0.030	0.864

## Data Availability

All data are in manuscripts.
